# Disparities in Substance Use Disorder Telehealth Services

**DOI:** 10.1001/jamanetworkopen.2024.59606

**Published:** 2025-02-12

**Authors:** Lauryn Saxe Walker, Manying Cui, Jonathan Cantor, Melody Craff, Cheryl L. Damberg, John N. Mafi, Katherine L. Kahn

**Affiliations:** 1 Virginia Center for Health Innovation, Richmond; 2 Division of General Internal Medicine & Health Services Research, David Geffen School of Medicine at UCLA, University of California, Los Angeles; 3 RAND, Santa Monica, California; 4 MedInsight, Milliman Inc, Seattle, Washington

## Abstract

This cross-sectional study evaluates whether telehealth utilization is associated with changes in payer-based and rural-urban disparities in substance use disorder treatment.

## Introduction

Telehealth has become a critical modality for substance use disorder treatment (SUDT) and focus of state and federal policy.^1,2^ Telehealth offers the potential to decrease disparities in SUDT for Medicaid and rural populations by reducing challenges with transportation and workforce shortage areas.^3^ Using nationwide claims data throughout the COVID-19 public health emergency (PHE), we examine telehealth’s association with changes in disparities in SUDT utilization across payers and rurality.

## Methods

We used the MedInsight Emerging Experience database, which includes Medicaid managed care, Medicare Advantage (MA), and commercial insurance claims across all 50 states.^4^ Medicare fee-for-service (FFS) claims are excluded from this analysis due to incomplete data on SUDT diagnoses and services. We constructed a retrospective rolling cohort of US adults aged 18 years or older, who were insured for at least 12 months during the study period (January 1, 2019, to June 30, 2023) (N = 16.2 million). This study was deemed exempt from UCLA institutional review board review. Informed consent was not required due to the use of deidentified data. This study followed the cross-sectional STROBE reporting guideline.

We calculated utilization for in-person, telehealth, and overall (telehealth + in-person) SUDT using the Milliman Health Cost Guidelines grouper.^5^ Telehealth services were identified through procedure and modifier codes and place of service (eAppendix in Supplement 1). We assessed proportional use of each SUDT modality compared with overall SUDT and compared average telehealth SUDT utilization per month with in-person SUDT. To adjust for enrollment volumes, we calculated average monthly SUDT utilization per 100 000 adults. All measures were stratified by payer and rurality. Analysis was conducted using Excel 365 version 2411 and Azure Databricks version 10.4 (Microsoft Corp). The MedIsight database served as a data warehouse and constructed the initial data extract for analysis. Precision of estimates is reported using 95% CIs.

## Results

Average monthly telehealth SUDT increased substantially for all populations between 2019 and 2023, from 44.6 to 10 974.3 services. Comparatively, in-person SUDT increased slightly, from 186 064 to 292 364 services.

**Figure 1.**
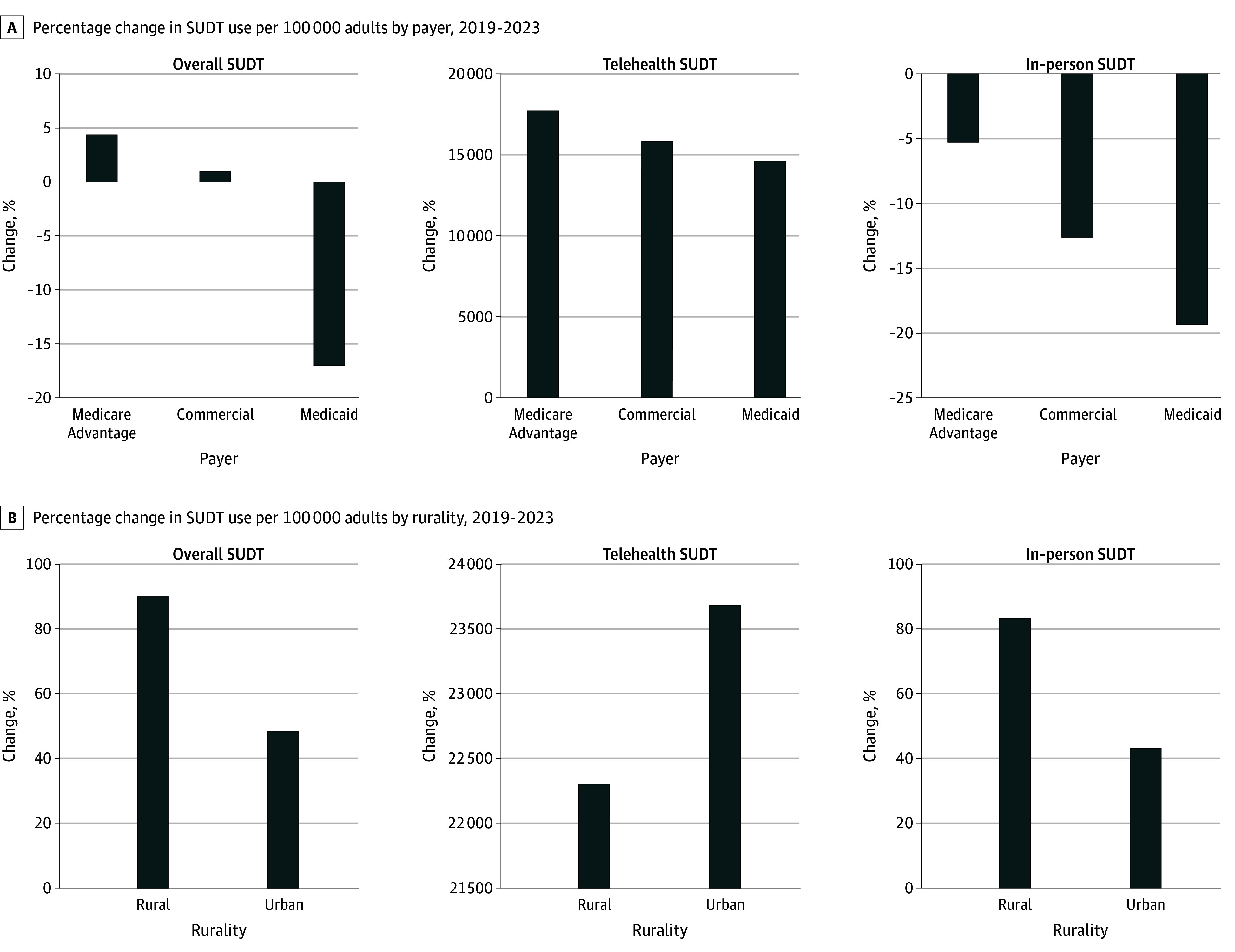
Percentage Change in Substance Use Disorder Treatment (SUDT) by Payer and Rurality, 2019 to 2023 A, Despite increases in telehealth SUDT across all payers, corresponding reductions in in-person SUDT resulted in overall decreases in SUDT use per 100 000 for individuals covered by Medicaid and overall increases for individuals covered by commercial insurers and Medicare Advantage. Adults who are dual eligible for Medicaid-Medicare are included as Medicaid members to create a single payer group for low-income individuals. B, All modalities of SUDT increased for both rural and urban individuals; however, overall SUDT increased for rural individuals to a greater extent than urban individuals. Rurality is determined by Rural-Urban Commuting Area Codes.

For overall SUDT, after accounting for health plan enrollment, Medicaid-covered individuals experienced a 17% decrease in average monthly SUDT per 100 000 adults (11 791 to 9788 services per 100 000). In contrast, MA-enrolled and commercially insured individuals experienced a 4% and 1% increase, respectively (Figure 1).

**Figure 2.**
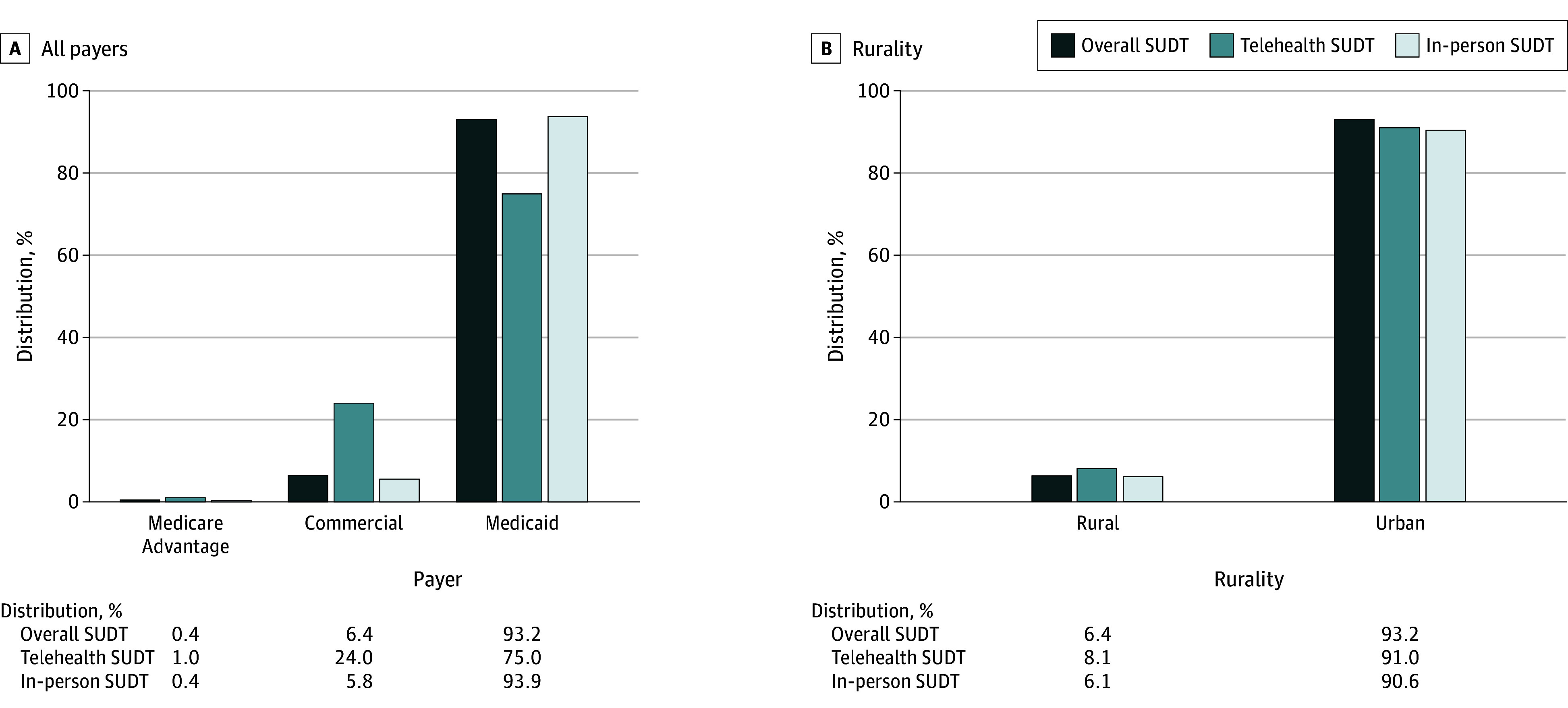
Distribution of Payer and Rurality Populations Across Substance Use Disorder Treatment (SUDT) Modalities, 2023 A, As opposed to telehealth SUDT being proportional to overall SUDT utilization for any given payer, proportionality varied by payer. Specifically, Medicaid telehealth SUDT was disproportionately low (74.9% of all telehealth SUDT) compared with overall SUDT (93.0% of overall SUDT). In contrast, rural telehealth SUDT was proportionate to overall SUD (6.3% and 6.2%, respectively). Adults who are dual eligible for Medicaid-Medicare are included as Medicaid members to create a single payer group for low-income individuals. Rurality is determined by Rural-Urban Commuting Area Codes.

MA-enrolled and commercially insured individuals were disproportionately represented within telehealth SUDT utilization (Figure 2). As of 2023, MA and commercially insured individuals accounted for 1.0% (95% CI, 1.0%-1.1%) and 24.0% (95 % CI, 23.3%-24.6%) of telehealth SUDT, respectively, yet 0.4% (95% CI, 0.4%-0.4%) and 6.4% (95% CI, 6.1%-6.7%) of overall SUDT. Medicaid-covered individuals were underrepresented in telehealth SUDT, accounting for 75.0% (95% CI, 74.4%-75.6%) of telehealth SUDT utilization and 93.2% (95% CI, 92.9%-93.5%) of overall SUDT.

Rural individuals experienced an 89.9% increase in overall SUDT per 100 000 adults (1521 to 2889 services per 100 000), nearly twice the increase of 48.7% among urban individuals (2204 to 3278 services per 100 000) (Figure 1). With rural individuals accounting for 8.1% (95% CI, 7.9%-8.4%) of telehealth SUDT and 6.4% (95% CI, 6.1%-6.6%) of overall SUDT, no disproportionality in telehealth SUDT use was identified (Figure 2).

## Discussion

MA-enrolled and commercially insured individuals were disproportionately more likely to use telehealth, with overall SUDT increasing over time. Medicaid-covered individuals were underrepresented within telehealth SUDT utilization, with overall SUDT declining. This finding raises equity concerns for Medicaid populations. For rural individuals, telehealth may be reducing disparities, with overall SUDT increasing throughout the PHE to a greater extent than for urban individuals.

This study has limitations. Organizations voluntarily contribute to the Emerging Experience database; therefore, data may not generalize. However, the database reflects payer coverage consistent with US census demographics, excluding Medicare FFS.^4^ This study is limited to services resulting in claims, which excludes uninsured individuals and clinicians not accepting insurance. This study does not differentiate by substance or assess care quality.

The findings of this study suggest that telehealth SUDT shows promise in reducing utilization disparities for rural individuals but not Medicaid-covered individuals. As policymakers define best practices for telehealth SUDT, consideration should be given to disparities in telehealth SUDT utilization.^6^

## Notes

Supplement 1. **eAppendix.** Supplementary Methods

Supplement 2.
Data Sharing Statement
