# Supplementary material for: Disparities in Substance Use Disorder Telehealth Services
Source: JAMA Netw Open. 2025 Feb 12;8(2):e2459606. doi: 10.1001/jamanetworkopen.2024.59606 (PMC11822531; doi:10.1001/jamanetworkopen.2024.59606)
Supplement: Supplement 1. — eAppendix. Supplementary Methods [file jamanetwopen-e2459606-s001.pdf]

Supplemental Online Content

Walker LS, Cui M, Cantor J, et al. Disparities in substance use disorder telehealth services. *JAMA Netw Open.* 2025;8(2):e2459606.
doi:10.1001/jamanetworkopen.2024.59606

eAppendix. Supplementary Methods

This supplemental material has been provided by the authors to give readers additional information about their work.

© 2025 Walker LS et al. *JAMA Network Open*.

eAppendix. Supplementary Methods

This study followed the STROBE guidelines for a cross-sectional study. Precision of estimates is reported using 95% confidence intervals (CIs).

Defining SUDT

SUDT is defined using the Milliman Health Cost Guidelines grouper which categorizes claims based on a combination of Current Procedural Terminology (CPT)/ Healthcare Common Procedure Coding System (HCPCS) procedure and modifier codes, revenue codes, and diagnoses.

Examples of services categorized as SUD services per Milliman HCG (HCPCS codes) [1]:

- **G2011**: Alcohol and/or substance (other than tobacco) misuse structured assessment and brief intervention 5-14 minutes
- **G2068**: Initiation of medication for opioid use disorder in an outpatient setting (buprenorphine)
- **G2080:** Add-on, additional 30 minutes of counseling in a week of medication assisted treatment
- **G2086**: Initial month, office-based treatment for opioid use disorder, including development of the treatment plan, care coordination, individual therapy and group therapy and counseling; at least 70 minutes in the first calendar month

Defining telehealth

Telehealth services were identified through procedure and modifier codes and place of service. Specific codes used to define telehealth are presented below. Due to variation in state policies and COVID-19 pandemic flexibilities, some telehealth services may not be captured.

Code Type	Code
CPT/HCPCS	Telephonic: 99441, 99442, 99443, 98966, 98967, 98968<br><br>Virtual check-in: G2012, G2010<br><br>Online e-visit: 99421, 99422, 99423, G2061, G2062, G2063, 98970, 98971, 98972<br><br>Remote physiologic monitoring: 99453, 99457, 99458
Modifiers	95, G1, GT
Place of Service	02, 10

[1] Center for Medicare and Medicaid Services. Billing & Payment. Last updated, November 20, 2024. https://www.cms.gov/medicare/payment/opioid-treatment-program/billing-payment

© 2025 Walker LS et al. *JAMA Network Open*.
