# Supplementary material for: Disparities in Substance Use Disorder Telehealth Services
Source: JAMA Netw Open. 2025 Feb 12;8(2):e2459606. doi: 10.1001/jamanetworkopen.2024.59606 (PMC11822531; doi:10.1001/jamanetworkopen.2024.59606)
Supplement: Supplement 2. — Data Sharing Statement [file jamanetwopen-e2459606-s002.pdf]

Data Sharing Statement

Walker. Disparities in Substance Use Disorder Telehealth Services. *JAMA Netw Open*. Published February 12, 2025. doi:10.1001/jamanetworkopen.2024.59606

Data
Data available: No

Additional Information
Explanation for why data not available: The MedInsight Emerging Experience database used for this study is considered proprietary to Milliman MedInsight® and therefore cannot be shared.
